# The Effectiveness of Acupuncture in Prevention and Treatment of Postoperative Nausea and Vomiting - A Systematic Review and Meta-Analysis

**DOI:** 10.1371/journal.pone.0082474

**Published:** 2013-12-13

**Authors:** Kah Bik Cheong, Ji-ping Zhang, Yong Huang, Zhang-jin Zhang

**Affiliations:** 1 School of Traditional Chinese Medicine, Southern Medical University, Guangzhou, Guangdong, China; 2 School of Chinese Medicine, LKS Faculty of Medicine, The University of Hong Kong, Hong Kong SAR, China; Iran University of Medical Sciences, Islamic Republic of Iran

## Abstract

**Background:**

Acupuncture therapy for preventive and treatment of postoperative nausea and vomiting(PONV), a condition which commonly present after anaesthesia and surgery is a subject of growing interest.

**Objective:**

This paper included a systematic review and meta-analysis on the effect of different type of acupuncture and acupoint selection in PONV prevention and treatment.

**Methods:**

Randomised controlled trials(RCTs) comparing acupuncture with non-acupuncture treatment were identified from databases PubMed, Cochrane, EBSCO, Ovid, CNKI and Wanfangdata. Meta-analysis on eligible studies was performed using fixed-effects model with RevMan 5.2. Results were expressed as RR for dichotomous data, with 95%CI.

**Results:**

Thirty RCTs, 1276 patients (intervention) and 1258 patients (control) were identified. Meta-analysis showed that PC6 acupuncture significantly reduced the number of cases of early vomiting (postoperative 0-6h) (RR=0.36, 95%CI 0.19,0.71; P=0.003) and nausea (postoperative 0-24h) (RR=0.25, 95%CI 0.10,0.61; P=0.002), but not early nausea (postoperative 0-6h) (RR=0.64, 95%CI 0.34,1.19; P=0.150) and vomiting (postoperative 0-24h) (RR=0.82, 95%CI 0.48,1.38; P=0.450). PC6 acupressure significantly reduced the number of cases of nausea (RR=0.71, 95%CI 0.57,0.87; P=0.001) and vomiting (RR=0.62, 95%CI 0.49,0.80; P=0.000) at postoperative 0-24h. PC6 electro-acupoint stimulation significantly reduced the number of cases of nausea (RR=0.49, 95%CI 0.38,0.63; P<0.000) and vomiting (RR=0.50, 95%CI 0.36,0.70; P<0.000) at postoperative 0-24h. Stimulation of PC6 with other acupoint(s) significantly reduced the number of cases of nausea and vomiting (RR=0.29, 95%CI 0.17,0.49; P<0.000) at postoperative 0-24h. Stimulation of other acupoint(s)(non PC6) also significantly reduced the number of cases of nausea and vomiting (RR=0.63, 95%CI 0.49,0.81; P=0.000) at postoperative 0-24h. However, the quality of study was generally low in studies of PC6 combined with other acupoint(s) and other acupoint(s). Details of blinding were not reported in most reports.

**Conclusions:**

Besides PC6, PC6 combined with other acupoint(s) and other alternative acupoint(s) might be beneficial in prevention and treatment of PONV, the evidence justifies future high-quality studies.

## Introduction

Postoperative nausea and vomiting (PONV) is a condition commonly present after anaesthesia and surgery, with overall incidence of 40%-90%[1]. Despite the use of newer drugs, PONV within 24 hours still occurs in 25%-30% of patients[2].

Though self-limitating, PONV can cause significant morbidity including dehydration, electrolyte imbalance, suture tension and dehiscence, venous hypertension and bleeding, esophageal rupture, and life-threatening airway compromise, although the more severe complications are rare[3]. PONV increases medical cost. An episode of vomiting could prolong postanaesthetic care unit (PACU) stay by about 25min[4].

Type of anaesthesia, type of surgery and site of operation contribute to PONV occurrence rate. Breast and gynaecological surgeries presented the most frequent report of PONV in adults[1]. Operations associated with high incidence of vomiting in children include strabismus, adenotonsillectomy, hernia repair, orchidopexy and penile surgery[4]. Research also demonstrated higher PONV occurrence rate in patients under general anaesthesia[5].

Limited efficacy and side effects with antiemetics led to the use of alternative treatment[1]. Researches in various countries believe acupuncture improves the quality of patients’ life[2]. An inventory concerning 32,000 acupuncture consultations in UK revealed the most common adverse events of bleeding, needle pain and aggravation of symptoms, but none were serious[1]. Various type of acupuncture has been used in mainland China and abroad[2], but the most suitable method is yet to be confirmed. 

According to the theory of traditional Chinese medicine (TCM), surgery breaks the balanced state of the human body and disturbs the movement of both qi and blood[6], causes the stomach qi to reverse its direction and go upward, causing nausea and vomiting[6]. One of the PC6’s functions is to avoid the adverse flow of qi, thus is an effective acupoint in preventing nausea and vomiting[6].

Meta-analysis by Shiao SY and Dibble SL (2006)[7] showed additional effective meridian points included Korean hand points(K-K9, K-D2), bladder points(BL10, BL11, BL18-26), spleen points(SP4, SP6), stomach points(ST34, ST36, ST44), and others. Countries abroad found more than 30 meridians and acupoints effective for PONV treatment, though their specific use has not been thoroughly investigated[8].

Chu YC et al. 1998 found that prophylactic bilateral stimulation with noninvasive acuplaster at BL10, BL11 and GB34 in children significantly reduces vomiting after strabismus correction[9]. PC6 may act only on hollow organs while these acupoints are more related to the meridians of the eye[9].

Researchers gradually realised that PC6 may not be the only acupoint in PONV treatment[8]. Patients' diseases and specific symptoms should be considered for method of selection[8].

The timing of acupuncture intervenes has also been an argument. Dundee JW and Ghaly RG (1989)[10] demonstrated a significant reduction in PONV incidence following preoperative PC6 acupuncture. However, Weightman WM et al. (1987)[11], did not find similar effect in their studies. The former[10] gave a possible explanation in terms of the timing of acupuncture intervene. To be effective, it should be administrated before the emetic stimulus. Yang LC et al. (1993)[12], however, found that PC6 electro-acupuncture administered in the recovery room was effective in reducing postoperative emesis.

 This study is carried out to evaluate the efficacy of different type of acupuncture, acupoint selection, optimal timing, technique of intervention, side effects and used of rescue therapy in PONV in the recent years.

## Materials and Methods

A research protocol was drafted and approved by the faculty members. A copy was kept by the principal investigator.

Search criteria: We combined the following MeSH and text words with filters:

1. English phrase: postoperative, nausea and vomiting, acupuncture, acupoints, acupressure, transcutaneous electric nerve stimulation, electrical acupoint stimulation, electrical acustimulation, electroacustimulation, electro-acupuncture, auricular acupuncture, moxa, moxibustion, warm needle therapy, sticking therapy
2. Chinese phrase: 术后“shu hou”, 恶心“e xin”, 呕吐“ou tu”, 针刺“zhen ci”, 针灸“zhen jiu”, 电刺激“dian ciji ”, 穴位“xue wei”, 按压“an ya”, 指压“zhi ya”, 电针“dian zhen”, 耳针“er zhen”, 温针“wen zhen”, 艾条“ai tiao”, 艾灸“ai jiu”, 敷贴“fu tie”

Database: PubMed, Cochrane Controlled Trials Register (CCTR), EBSCO, OVID, CNKI, Wanfangdata.

Supplementary search: http://www.google.cn and http://www.clinicaltrials.gov; to search for articles which could not be assessed from the database via the university library website and to check for any left out trials.

Unpublished trials were not included.

Any uncertainties were clarified by contacting the respective corresponding authors via e-mails. 

### Selection criteria

***Inclusioncriteria:*** 1. randomised controlled clinical trials (RCTs); 2. patients underwent surgery regardless of age, gender, ethnic, type of anaesthesia or surgery; 3. all forms of acupuncture; 4. publications within 1986 to 30 Jun 2013, full text articles in English or Chinese.

#### Outcome measures

Primary outcomes: efficacy of different type of acupuncture and acupoint selection in prevention and treatment of PONV

Subgroups were divided according to the type of acupuncture (manual acupuncture, acupressure, electro-acupoint stimulation), acupoint (PC6, PC6 combined with other acupoint(s), other acupoint(s)) and time of PONV. 

Control group consisted of standard care, sham, medication or counseling. 

Complete prevention was defined as absence of nausea and vomiting within 0-6 h (early PONV), 6-24h (late PONV) and 0-24h for the whole operation.

Secondary outcomes: optimal timing, technique of intervention, side effects and used of rescue therapy

***Exclusioncriteria:*** 1. non-randomised trials; 2. non clinical trials; 3. patients with other co-existing acute or chronic illness; 4. patients nausea and vomiting before operation; 5. patients taking anti-emetics medication before operation; 6. articles not in English or Chinese; 7. duplicate articles; 8. articles which data analysis did not fulfill protocol criteria.

### Data collection and analysis

Evaluation was performed independently by 2 authors (KBC & JPZ). Relevant, full articles were sorted and cross-examined. Any discrepancies were discussed or further evaluated by a 3rd author (YH). Data was collected using MS Excel 2010 which included the title of journals, author(s), year of publication, type of randomisation, type and duration of anaesthesia and surgery, type of intervention, sample size, details of participants, timing and technique of intervention, needle retention, depth of needle insertion, frequency and duration of intervention, results, conclusion, side effects and use of rescue therapy.

**Figure 1:**
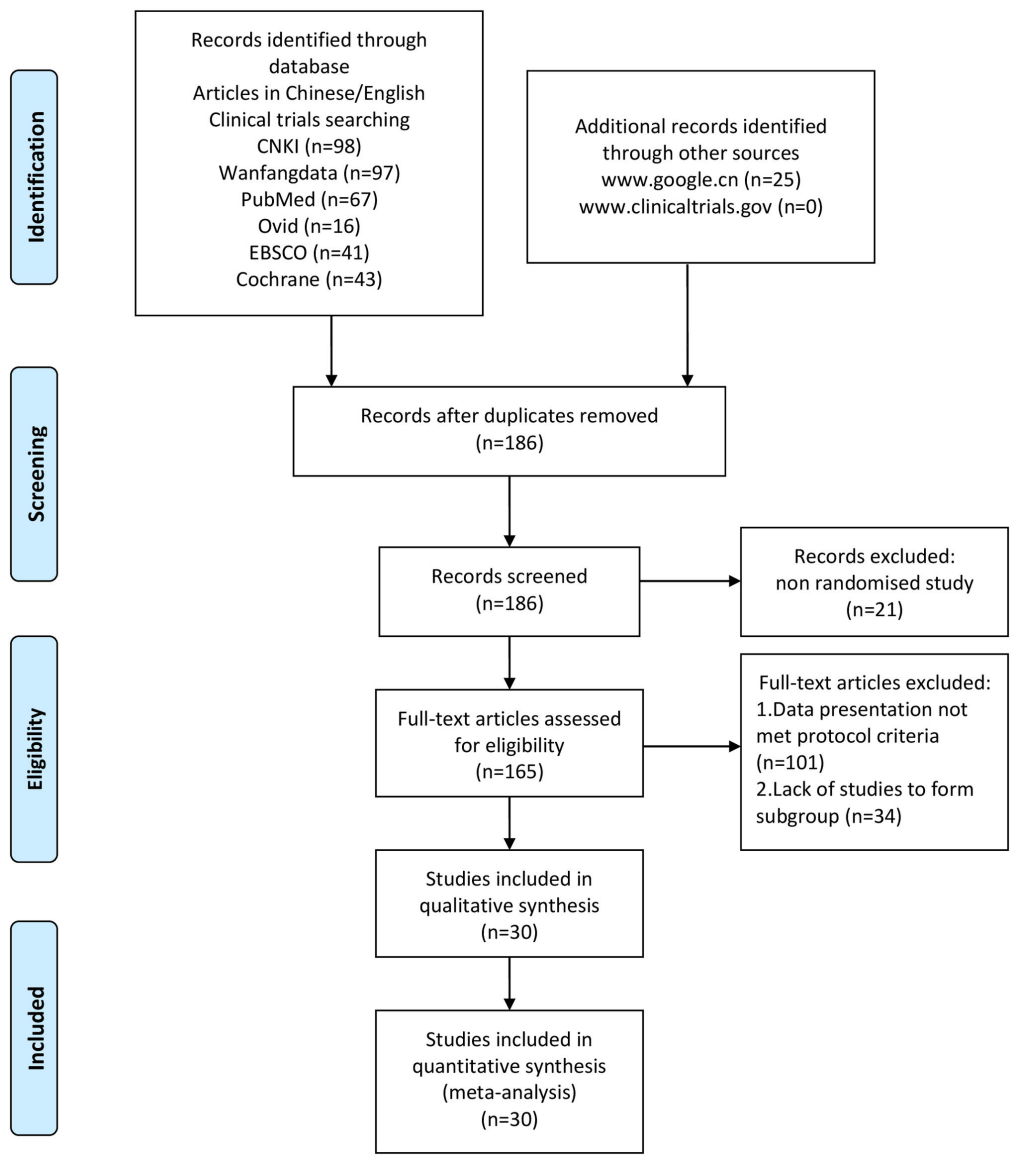
PRISMA 2009 Flow Diagram for data collection and analysis.

All trials satisfying the inclusion criteria were included in initial analysis (Figure 1). Trials whose protocols varied significantly from others were excluded.

Meta-analysis was performed using fixed-effects model with RevMan 5.2. Analysis was presented as RR (relative risk) for dichotomous data and 95%CI with P<0.05 as significant level. I^2^ values of 25%, 50%, and 75% represent low, moderate and high heterogeneity. Funnel plots were performed to check the existence of bias (outcome level). If heterogeneity showed P<0.1 or I^2^>50, sensitivity analysis were carried out; any outlier would be examined the cause of differences.

Quality of studies was assessed using GRADE profiler version 3.6. Items evaluated included:

1 risk of bias/study limitations(study level), inconsistency, indirectness, imprecision and publication bias (downgrade quality of evidence)
2 large effect, plausible confounding and dose response gradient (upgrade quality of evidence

According to GRADE Working Group grades of evidence[13], quality of studies was graded as high, moderate, low or very low:

1 High quality: Further research is very unlikely to change our confidence in the estimate of effect.
2 Moderate quality: Further research is likely to have an important impact on our confidence in the estimate of effect and may change the estimate.
3 Low quality: Further research is very likely to have an important impact on our confidence in the estimate of effect and is likely to change the estimate.
4 Very low quality: We are very uncertain about the estimate.

All trials were evaluated using CONSORT[14] and STRICTA[15] for TCM according to the standard guideline. Items evaluated included title and abstract, introduction, methods, discussion and other information for CONSORT; acupuncture rationale, details of needling, treatment regimen, other components of treatment, practitioner background and control or comparator interventions for STRICTA. 

## Results

Data was summarised in Table S1A-C. Of the 186 studies reviewed, finally 30 studies met the inclusion criteria for meta-analysis: 16(53.33%) on PC6[16-31], 6(20.00%) on PC6 combined with other acupoint(s)[32-37] and 8(26.67%) on other acupoint(s) (including auricular acupoints)[38-45].

All 16 studies on PC6 served as prevention. Of the 6 studies on PC6 combined with other acupoint(s), interventions were served as analgesic and prevention[32,35,36], prevention[33,37], and treatment[34]. Of the 8 studies on other acupoint(s) (3 on auricular acupuncture[42,45]/acupressure[41]), interventions were served as prevention[38,41,43-45], prevention and treatment[39], analgesic and prevention[40,42].

Of the 30 studies, 2(6.67%) were performed under i/v anaesthesia[16,25], 15(50.00%)[17,19,20,22,24,27,29-32,37,38,41,42,44] under general anaesthesia, 3(10.00%) under infusion-inhalation anesthesia (PCA)[18,21,26], 2(6.67%) under spinal anaesthesia[23,28], 4(13.33%) under epidural anaesthesia[35,36,39,45] and 2(6.67%) under local anaesthesia[40,43]. Another 2(6.67%) did not report the type of anaesthesia used[33,34]. 

One study on PC6[20] and 1 on other acupoint(s)[41] were based on paediatric population while the remaining were on adults.

### Type of acupuncture and acupoint selection

#### PC6 acupuncture

Postoperative nausea: 4 studies, 281 participants, were divided into subgroups according to the time of PONV.

**Figure 2:**
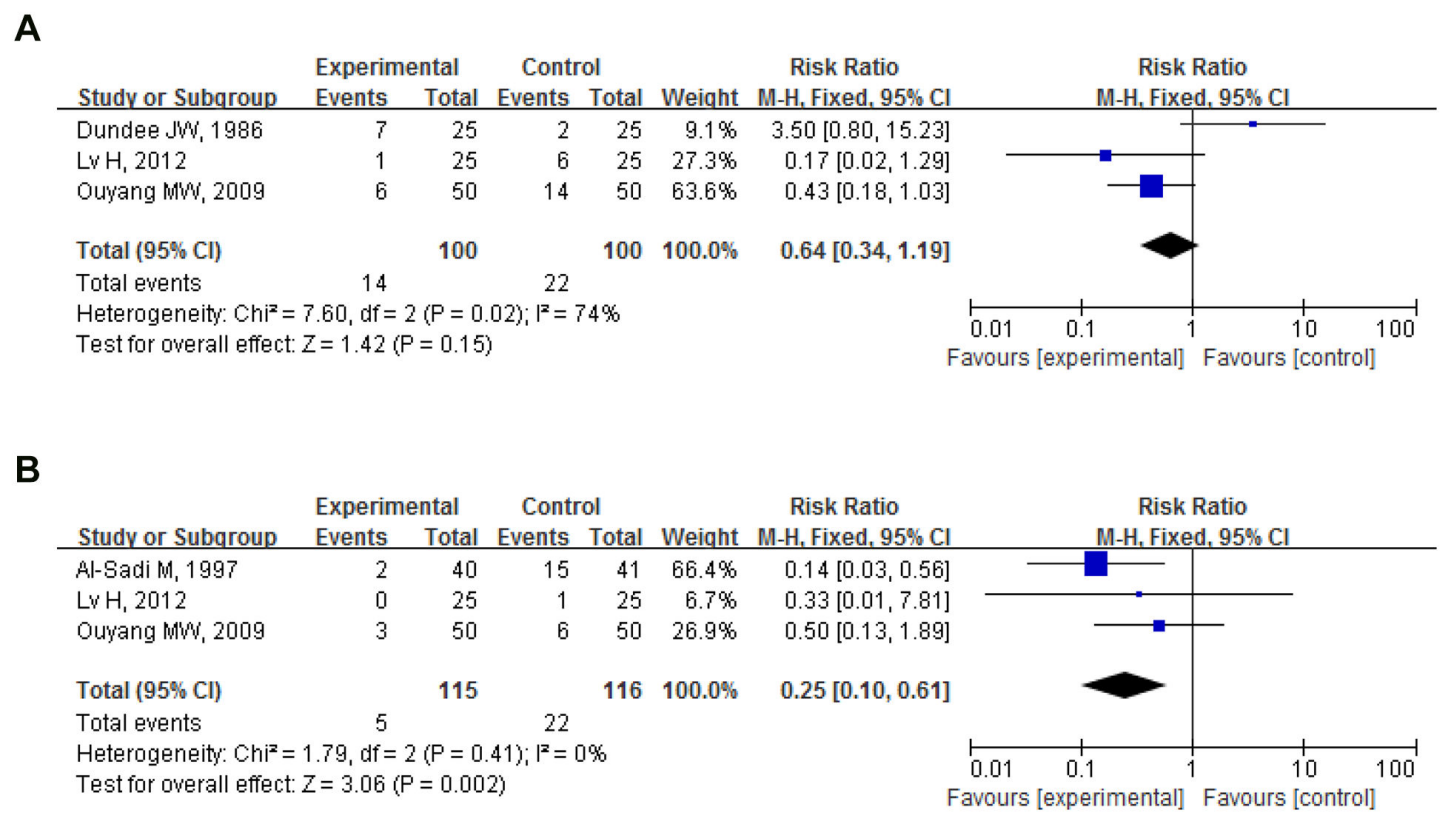
PC6 acupuncture vs. **no acupuncture (postoperative nausea)**. (**A**) Postoperative nausea (postoperative 0-6h). (**B**) Postoperative nausea (postoperative 0-24h).

Postoperative nausea 0-6h (early postoperative nausea): Proportion of nausea in 3 pooled trials (200 participants) was 14%(14/100) for PC6 acupuncture and 22%(22/100) for control (no acupuncture). Pooled RR was 0.64(0.34,1.19); P=0.150 with no significant difference between the 2 groups[16-18] (Figure 2A)

Postoperative nausea 0-24h: Proportion of nausea in 3 pooled trials (231 participants) was 4.35%(5/115) for PC6 acupuncture and 18.96%(22/116) for control (no acupuncture). Pooled RR was 0.25(0.10,0.61); P=0.002. PC6 acupuncture significantly reduced the number of cases of nausea[17-19] (Figure 2B)

Postoperative vomiting: 5 studies, 326 participants were divided into subgroups according to the time of PONV.

**Figure 3:**
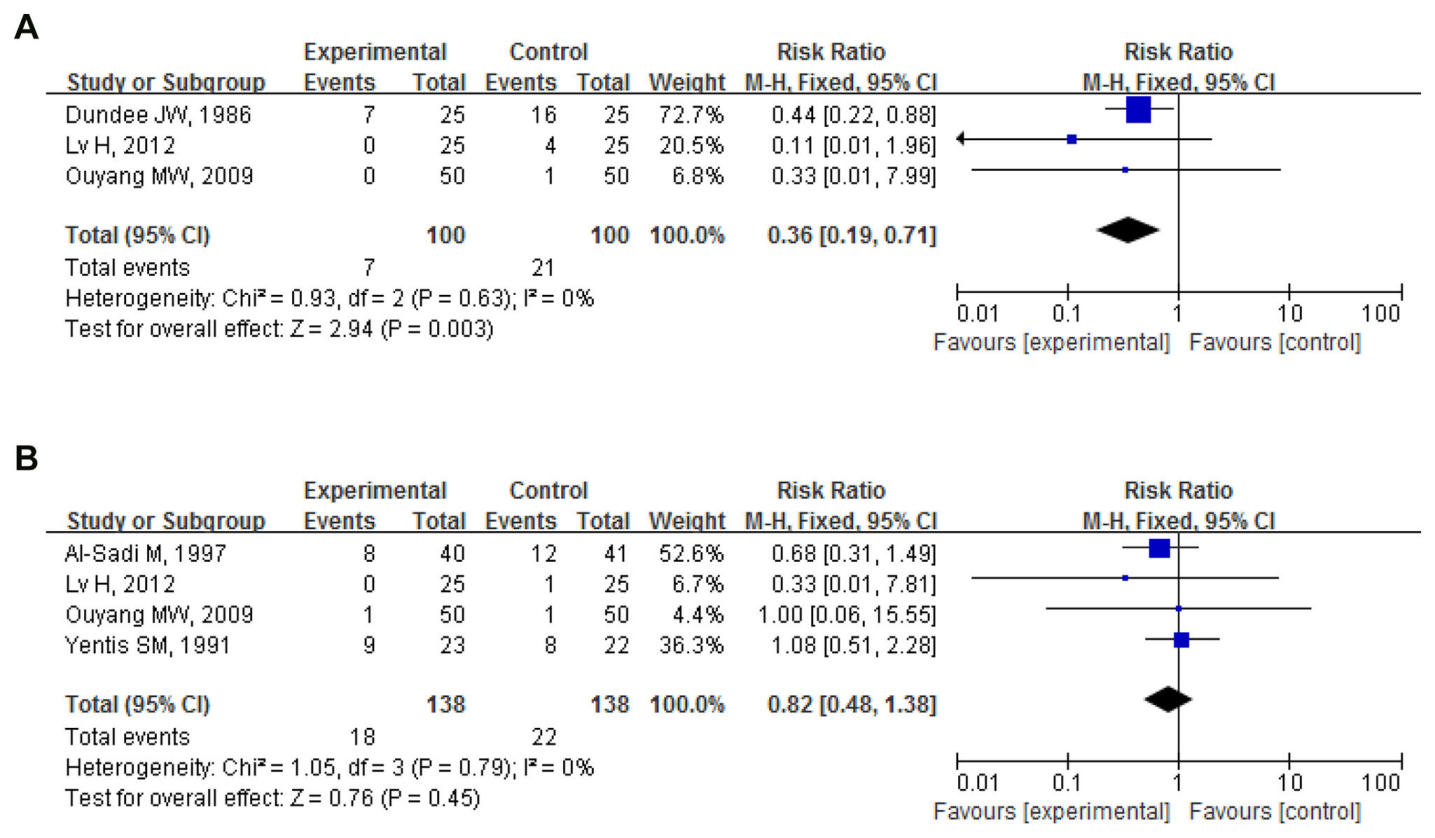
PC6 acupuncture vs. no acupuncture (postoperative vomiting). (**A**) Postoperative vomiting (postoperative 0-6h). (**B**) Postoperative vomiting (postoperative 0-24h).

Postoperative vomiting 0-6h (early postoperative vomiting): Proportion of vomiting in 3 pooled trials (200 participants) was 7.00%(7/100) for PC6 acupuncture and 21.00%(21/100) for control (no acupuncture). Pooled RR was 0.36(0.19,0.71); P=0.003. PC6 acupuncture significantly reduced the number of cases of vomiting[16-18] (Figure 3A.)

Postoperative vomiting 0-24h: Proportion of vomiting in 4 pooled trials (276 participants) was (13.04%)18/138 for PC6 acupuncture and 15.94%(22/138) for control (no acupuncture). Pooled RR was 0.82(0.48,1.38); P=0.450 with no significant difference between the 2 groups[17-20] (Figure 3B).

#### PC6 acupressure

**Figure 4:**
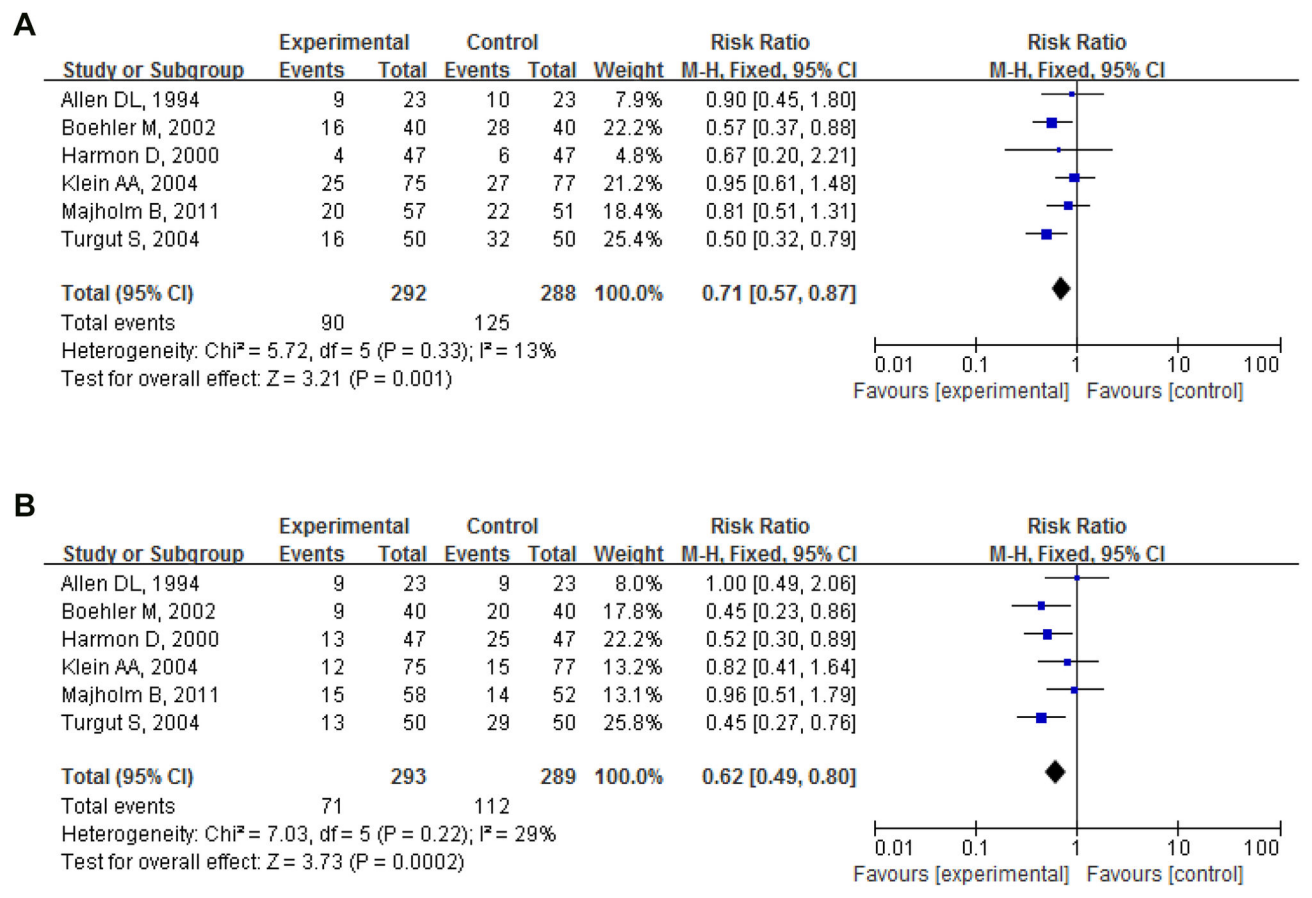
PC6 acupressure vs. sham (postoperative 0-24h). (**A**) Postoperative nausea. (**B**) Postoperative vomiting.

Postoperative nausea 0-24h: Proportion of nausea in 6 pooled trials (580 participants) was 30.82%(90/292) for PC6 acupressure and 43.40%(125/288) for sham control. Pooled RR was 0.71(0.57,0.87); P=0.001. PC6 acupressure significantly reduced the number of cases of postoperative nausea compared to sham group[21-26] (Figure 4A).

Postoperative vomiting 0-24h: Proportion of vomiting in 6 pooled trials (582 participants) was 24.23%(71/293) for PC-6 acupressure and 38.75%(112/289) for sham control. Pooled RR was 0.62(0.49,0.80); P=0.000. PC6 acupressure significantly reduced the number of cases of postoperative vomiting compared to sham group[21-26] (Figure 4B).

#### PC6 electro-acupoint stimulation

**Figure 5:**
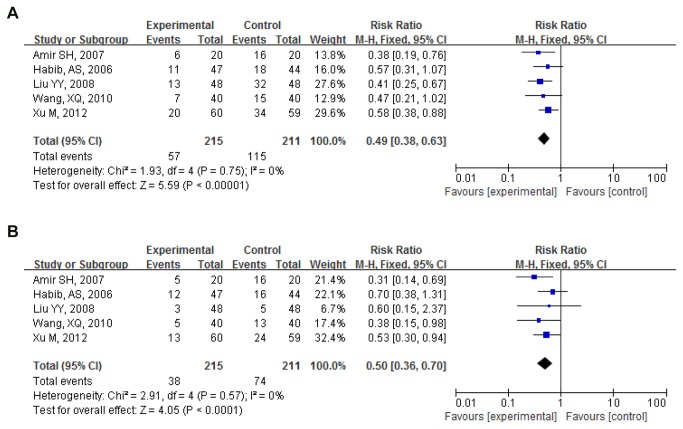
PC6 electro-acupoint stimulation vs. sham (postoperative 0-24h). (**A**) Postoperative nausea. (**B**) Postoperative vomiting.

Postoperative nausea 0-24h: Proportion of nausea in 5 pooled trials (426 participants) was 26.51%(57/215) for PC6 electro-acupoint stimulation and 54.50%(115/211) for sham control. Pooled RR was 0.49(0.38,0.63); P<0.000. PC6 electro-acupoint stimulation significantly reduced the number of cases of postoperative nausea compared to sham group[27-31] (Figure 5A).

Postoperative vomiting 0-24h: Proportion of vomiting in 5 pooled trials (426 participants) was 17.67%(38/215) for PC6 electro-acupoint stimulation and 35.07%(74/211) for sham control. Pooled RR was 0.50(0.36,0.70); P<0.000. PC6 electro-acupoint stimulation significantly reduced the number of cases of postoperative vomiting compared to sham group[27-31] (Figure 5B).

**Figure 6:**
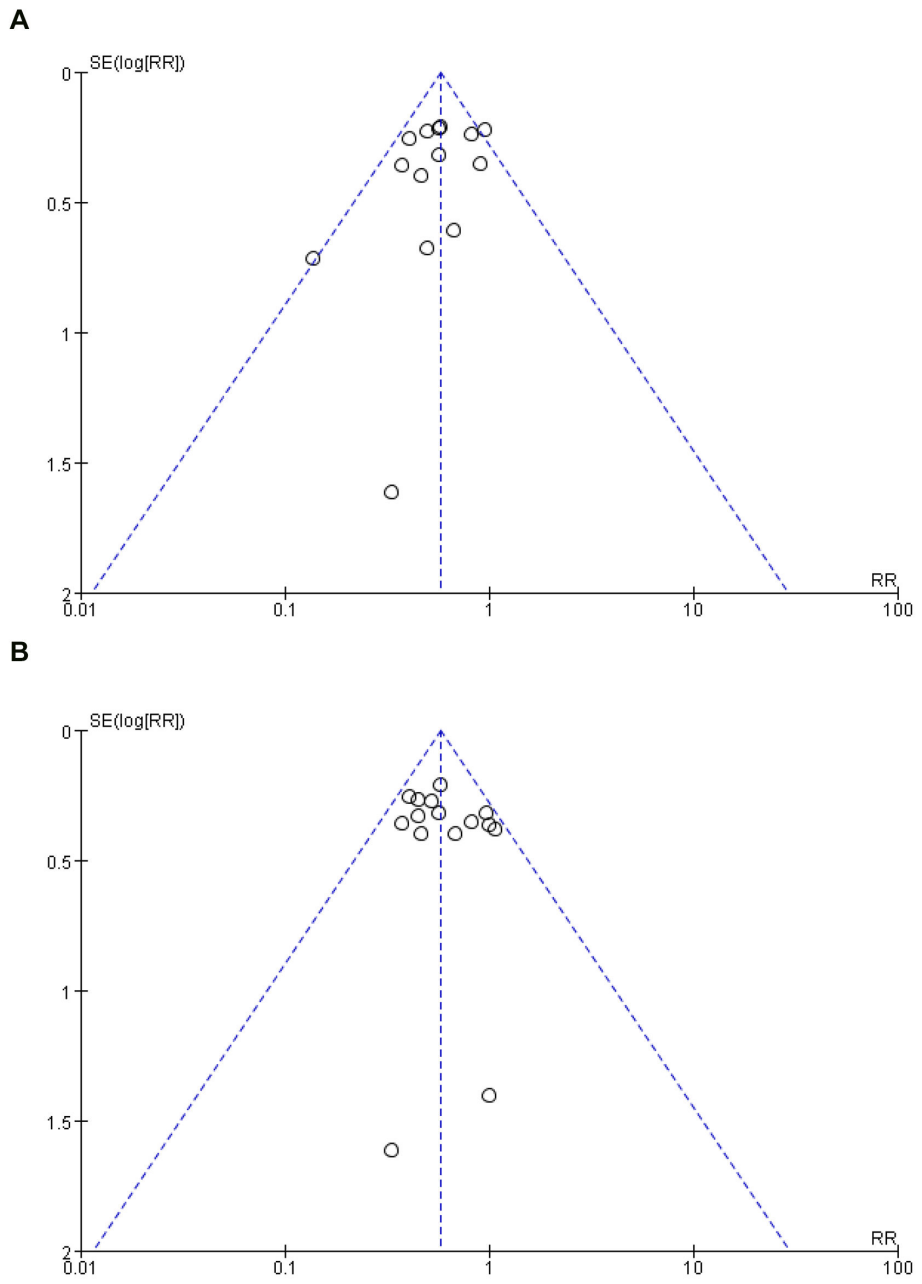
Funnel plot for PC6 acupoint vs. control (postoperative 0-24h). (**A**) Postoperative nausea. (**B**) Postoperative vomiting.

Funnel plots were shown in Figure 6.

#### PC-6 combined with other acupoint(s)

**Figure 7:**
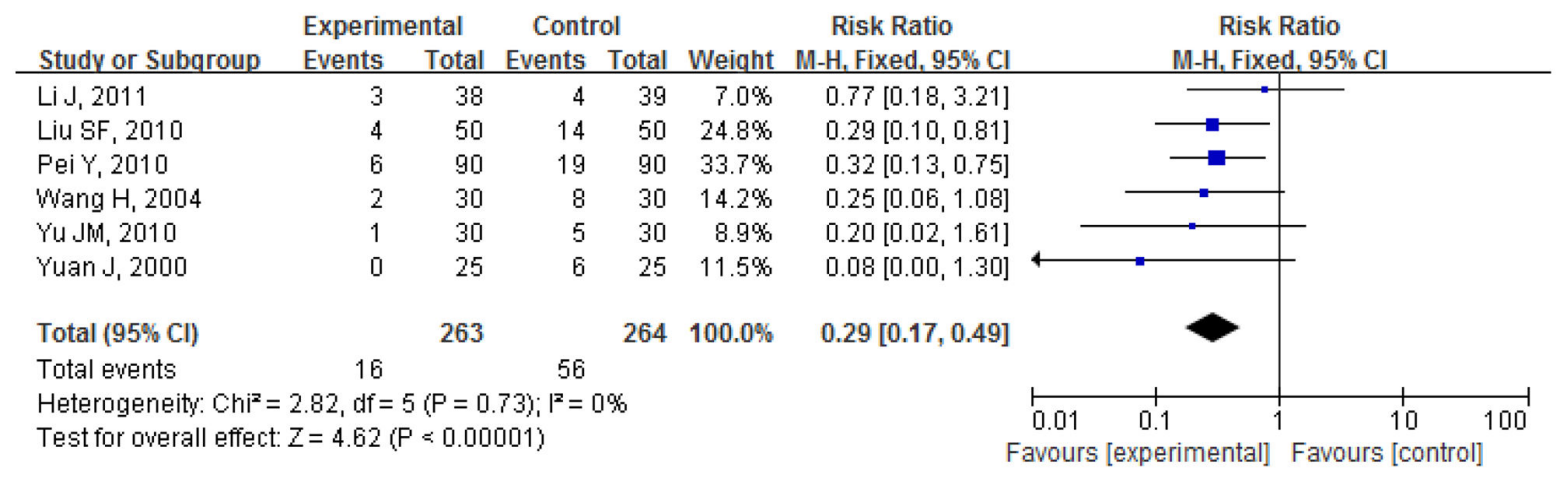
PC6 combined with other acupoint(s) vs. **control for PONV (postoperative 0-24h)**.

Postoperative nausea and vomiting 0-24h: Proportion of PONV in 6 pooled trials (527 participants) was 6.08%(16/263) for intervention group and 21.21%(56/264) for control group. Pooled RR was 0.29(0.17,0.49); P<0.000. Intervention group significantly reduced the number of cases of PONV compared to control group[32-37] (Figure 7). 

**Figure 8:**
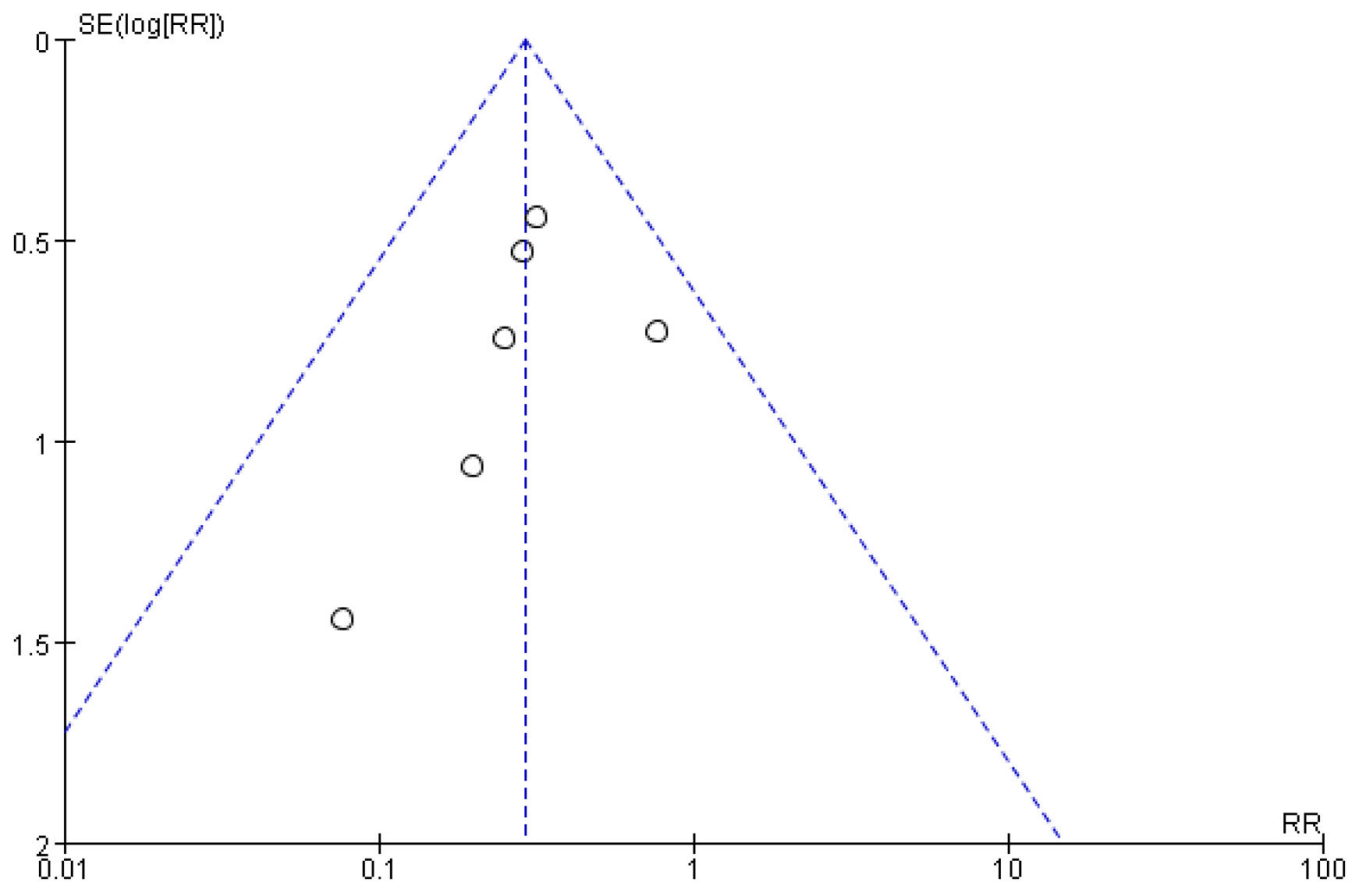
Funnel plot for PC6 combined with other acupoint(s) vs. control for PONV (postoperative 0-24h).

Funnel plot were shown in Figure 8.

#### Other acupoint(s)

**Figure 9:**
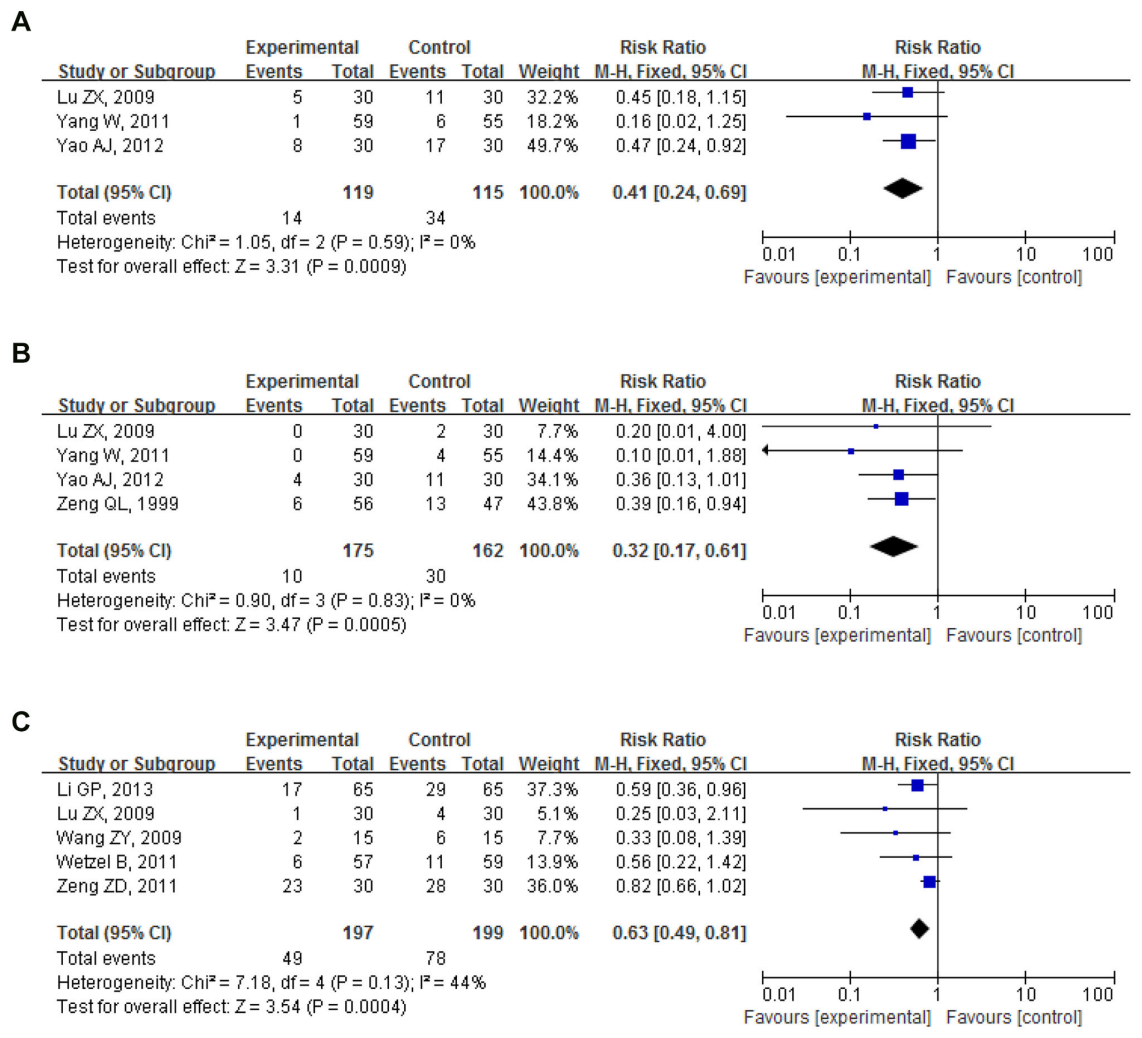
Other acupoint(s) (including auricular acupoints) vs. control (postoperative 0-24h). (**A**) Postoperative nausea. (**B**) Postoperative vomiting. (**C**) Postoperative nausea and vomiting.

Postoperative nausea 0-24h: Proportion of nausea in 3 trials (234 participants) was 11.76%(14/119) for intervention group and 29.56%(34/115) for control group . Pooled RR was 0.41(0.24,0.69); P=0.001. Intervention group significantly reduced the number of cases of postoperative nausea compared to control group[38-40] (Figure 9A).

Postoperative vomiting 0-24h: Proportion of vomiting in 4 trials (337 participants) was 5.71%(10/175) for intervention group and 18.52%(30/162) for control group. Pooled RR was 0.32(0.17,0.61); P=0.000. Intervention group significantly reduced the number of cases of postoperative vomiting compared to control group[38-41] (Figure 9B).

Postoperative nausea and vomiting 0-24h: Proportion of PONV in 5 trials (396 participants) was 24.87%(49/197) for intervention group and 39.20%(78/199) for control group. Pooled RR was 0.63(0.49,0.81); P=0.000. Intervention group significantly reduced the number of cases of PONV compared to control group[38,42-45] (Figure 9C).

**Figure 10:**
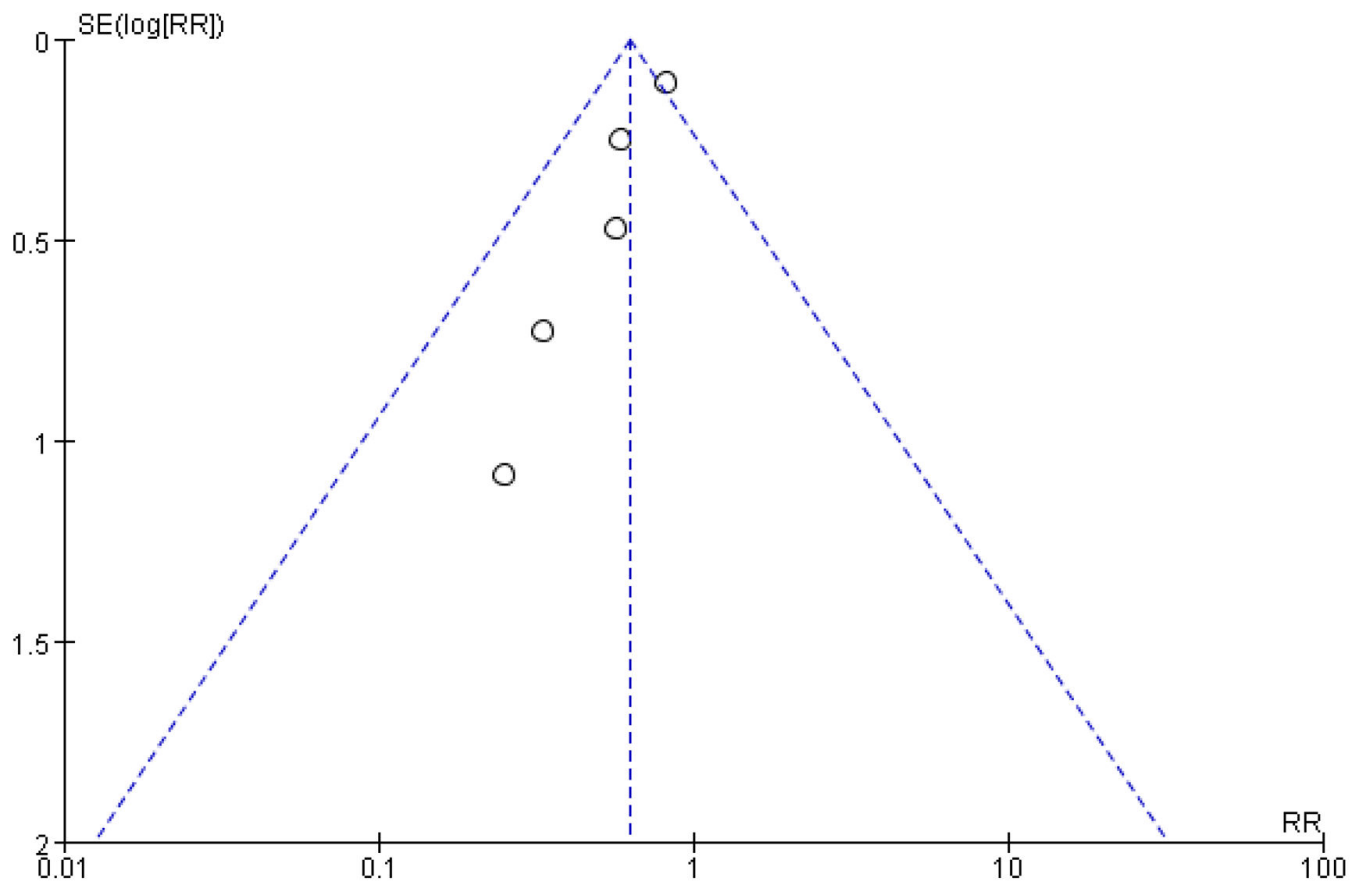
Funnel plot for other acupoint(s) vs. control for PONV (postoperative 0-24h).

Funnel plot were shown in Figure 10.

Results of meta-analysis for all subgroups showed low to moderate heterogeneity, with P>0.1 and I^2^<50 (Figure 2-5,7,9). No bias was demonstrated (Funnel plots, Figure 6,8,10). 

### Optimal timing and technique of intervention

#### Timing of intervention

For PC6 acupuncture, manual needling was administered before[18] and after induction of anaesthesia[16,19,20]; and postoperatively[17]. All studies in PC6 acupressure intervened prior to induction of anaesthesia[21-26]. This is similar for PC6 electro-acupoint stimulation[27-31]. 

For PC6 combined with other acupoint(s), intervention were carried out before[32,36] and during induction of anaesthesia[35]; and during operation[33,34,37].

For other acupoint(s), intervention were performed before[43] and after induction of anaesthesia[39]; preoperatively[40,42,43,45] and postoperatively[38,44].

#### Duration of needle retention/intervention

Time of needle retention for PC6 acupuncture varied from 5min[16,20], 30min[17] to whole duration of surgery[18,19]. PC6 acupressure was maintained for at least 24h[22,24,26]. Electro-acupoint stimulation was performed for 20min[27] or (30min[30,31] to 60min[28,29]) before induction of anaesthesia until 6h[30] to end of surgery[29] or 24 h[27,28,31] postoperatively.

For PC6 combined with other acupoint(s), in a study with manual acupuncture, needle was kept for every 7-8min until end of surgery[37], while for 30min[32] and 5-10 min[33] in transcutaneous electrical acupoint stimulation (TEAS).

For other acupoint(s), in 1 study, cupping therapy was applied for 10min at postoperative 6h and 24h[38]; in another study electro-acupuncture was performed for 25min, followed by acupoint injection bd[39]; in a study, auricular acupressure was performed for 1-3min for 2-3 times during surgery, followed by 3-4 times daily post-surgery[41]; in 1 study acupoint massage was performed for 10-15min every 4-6h[44]; and in another study auricular acupuncture was applied every 30min and kept until end of surgery[45].

#### Technique of intervention

Technique used in PC6 acupuncture included rotating, reinforcing-reducing[17,18] and rotating[19]. For PC6 acupressure “SeaBand”[21], “SeaBand” with beads[23,24,26] and “Vital-Band”[25] were used. Korean Hand acupressure used 2-mm diameter acupressure seeds[22]. For PC6 electro-acupoint stimulation, needling[27]; “active ReliefBand”[28]; surface[29] and (HANS) electrode[30,31] were used for stimulation. Electrical stimulation varied, with 4Hz[27] to 2-100Hz alternating waveform[29-31]. Reported current included 0.5-4mA, 50ms with conventional peripheral nerve stimulator (PNS) train-of-four (TOF) mode[29] and 2mA with HANS dual-channel unit[31].

For PC6 combined with other acupoint(s), technique used included TEAS at 2Hz/100Hz, 5-10mA[32], TEAS with relaxation therapy[33], acupoint injection[34], continuous electrical stimulation at 50-100Hz[35], electro-acupuncture at 16-50Hz, 10-15mA with HANS electrode[36] and manual acupuncture with rotating, reinforcing-reducing technique using filiform needles[37].

For other acupoint(s), cupping therapy[38], electro-acupuncture(10-50Hz, 1-2mA) with acupoint injection[39], catgut embedment[40], auricular acupressure (plaster therapy with Vaccaria seed)[41], auricular acupuncture[42,45], acupoint injection[43] and acupoint massage[44] had been used.

#### Acupoints (unilateral/bilateral)

Three studies in PC6 acupuncture intervened bilaterally[17-19] while one at left PC6[20]. For PC6 acupressure, intervention was performed at dominant wrist[21]; right[23] and bilateral PC6[24,26]. One study applied Korean Hand acupressure at bilateral K-K9[22]. Another study intervened at PC6 ipsilateral to the site of anaesthesia[25]. For PC6 electro-acupoint stimulation, “ReliefBand” and HANS electrode was applied to the dominant hand[28,31], and right PC6[30]. Surface electrode was applied to left PC6 in 1 study[29].

For PC6 combined with other acupoint(s), manual acupuncture was performed at bilateral PC6, LI4, BL10, GB34, ST36, SP4, CV12, with supplementary acupoints LV3, SP6, SP9 and ST40[37], TEAS at bilateral LI4 and PC6[33], electro-acupuncture at bilateral PC6, ST36 and LI4[35,36] and acupoint injection at bilateral PC6 and ST36[34].

For other acupoint(s), electro-acupuncture 10-50Hz was performed at bilateral LI4 with acupoint injection at bilateral ST36[39], catgut embedment at bilateral BL57[40], bilateral auricular acupressure at CO13, C04, AT(brain) and TF4[41], auricular acupuncture at MA-AH4(AH5), MA-TF1(TF4), MA-IC1(CO14) ipsilateral to the surgery site[42], acupoint injection at bilateral ST36[43], bilateral ST36 acupoint massage[44] and right auricular acupuncture at TF4, AT(brain), CO18, with supplementary acupoint at TF5 and TF(Uterus)[45]. 

#### Needle size

For PC6 acupuncture, needles used included 0.18mm and 0.20mm diameter[19,20] and 1-2cm, 30 steel wire gauge stainless steel[16]. For PC6 electro-acupoint stimulation, 1 study reported the use of (0.25 x 30)mm Serin no 5 Japan needles[27].

For PC6 combined with other acupoint(s), 1 study used no 1, 1.5 inch in length filiform needles for manual acupuncture[37].

For other acupoint(s), 1 study used auricular acupuncture needles size 0.22mm in diameter, 1.5mm in length[42], another study used disposable pinhead (0.90 x 38)mm and acupuncture needles of (0.30 x 50)mm for catgut embedment[40].

#### Depth of needle insertion

For PC6 acupuncture, depth of needle insertion reported included 5mm[17-19] and 1cm[16]. For PC6 combined with other acupoint(s), 1 study reported needle insertion of 0.8-1 inch[37]. For other acupoint(s), a study reported catgut embedment of 1.0-1.5cm[40].

### Side effects

Of the 30 studies, 10(33.33%) reported no side effects. One study(3.33%) with acupressure wristbands and sham, reported redness, swelling, tenderness and paraesthesia of wrist and hand in approximately 1/3 of patients. The local side effects caused by the acupressure wristband were equally distributed between PC6 stimulation and sham[25]. Another study with acupressure band reported swelling and erythema of the treated hand, where patient finally excluded from the study[26]. A study on electro-acupuncture reported local complication of erythema in 15% of cases[27]. Two studies(6.67%) reported no major side effects[23,37]. The remaining 15(50.00%) studies did not report whether there were any side effects in their findings.

### Use of rescue anti-emetics

Of the 30 studies, 15(50.00%) reported use of anti-emetics, while 11(36.67%) reported comparison between the intervention and control group. Significant differences were noted in 4(13.33%) studies[23,26,27,29], one with Metoclopramide 10mg i/v[26], another three with Ondansetron 4mg i/v[23,27,29].

Subset analysis by gender in 1 study[24] with Dimenhydrinate 50mg i/v showed that acupressure group female patients required less antiemetic than control. However, no significant difference was noted in male patients.

No significant differences were reported in 5(16.67%) studies[24,25,28,30,31] with Dimenhydrinate 50mg i/v[24], Ondansetron 4mg i/v[28] and Metoclopramide 10mg i/v[30] and i/m[31].

### Quality evaluation

#### GRADE

Of the 30 studies (Table S1A-C), 4(13.33%) from PC6 demonstrated high quality of evidence[19,23,28,31] which involved manual acupuncture[19], acupressure[23] and TEAS[28,31] conducted in UK[19], Ireland[23], USA[28] and China[31]. Nine studies in PC6 showed moderate quality of evidence[18,20,22,24-27,29,30] while three showed low quality[16,17,21]. 

All studies in PC6 combined with other acupoint(s) showed low quality of evidence[32-37]. One study on other acupoint(s) (conducted in German) demonstrated moderate quality[42] while the remaining showed low quality[38-41,43-45].

Moderate quality of evidence was mainly due to precision not reported in the study outcomes while low quality of evidence was due to study not blinded and precision not reported in the study outcomes.

#### CONSORT and STRICTA for TCM

**Figure 11:**
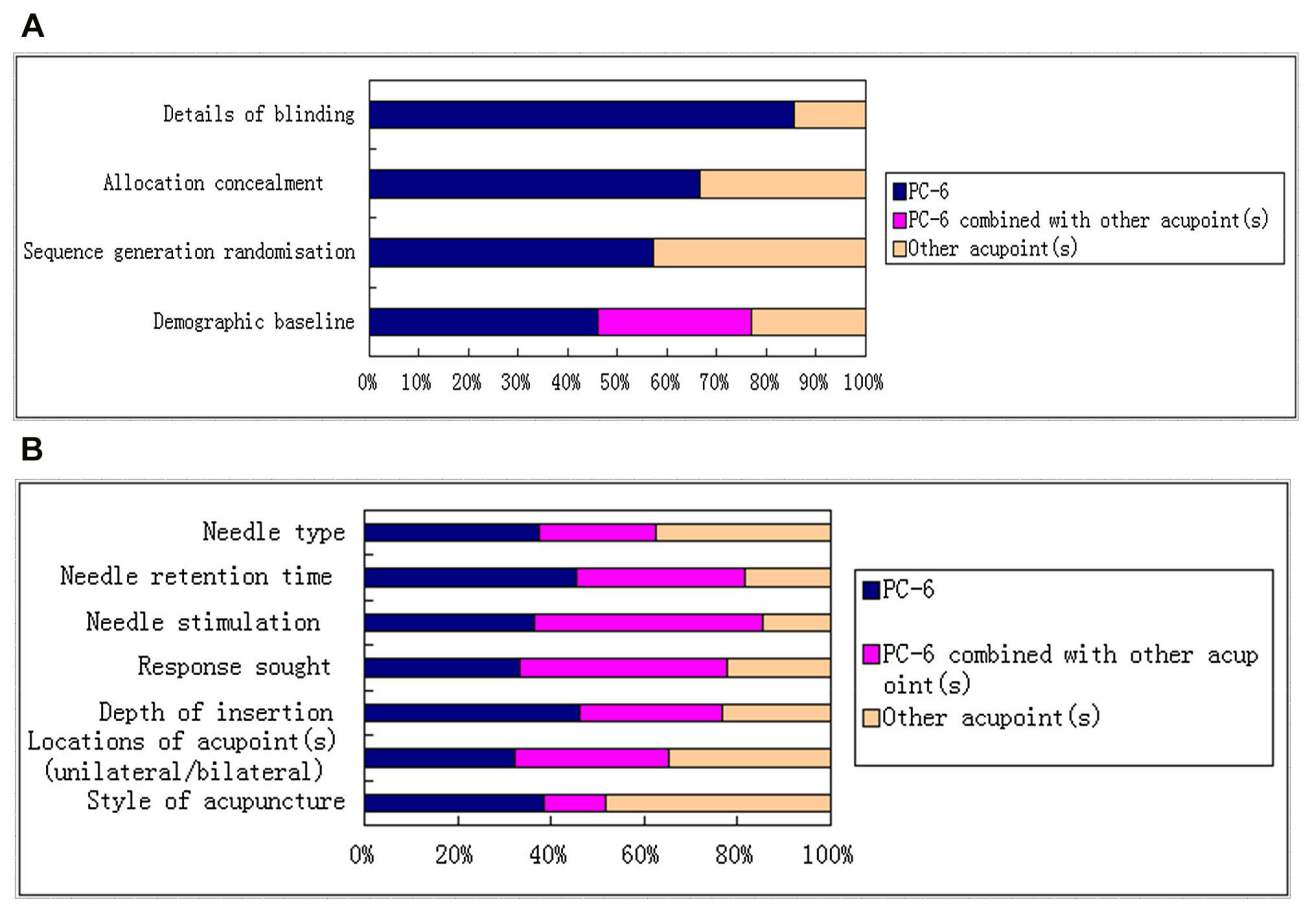
Quality assessment graph evaluated with CONSORT and STRICTA for TCM. (**A**) Percentage of important items reported (evaluated with CONSORT). (**B**) Percentage of important items reported (evaluated with STRICTA).

CONSORT: of the 30 studies, 18(60.00%) reported demographic baseline, 11(36.67%) reported sequence generalization randomisation, 5(16.67%) reported allocation concealment, 13(43.33%) reported details of blinding (Figure 11A). 

STRICTA: Of the 30 studies, 14(46.67%) reported the style of acupuncture, 25(83.33%) reported acupoint locations, 6(20.00%) reported depth of needle insertion, 11(36.67%) reported response sought, 17(56.67%) reported needle stimulation, 15(50.00%) reported duration of needle retention and 7(23.33%) reported needle type (Figure 11B).

## Discussion

### Type of acupuncture and acupoint selection

#### Type of acupuncture

For prevention of nausea (postoperative 0-24h), PC6 acupuncture vs. no acupuncture had the lowest pooled RR, followed by PC6 electro-acupoint stimulation vs. sham and PC6 acupressure vs. sham. PC6 acupuncture vs. no acupuncture seemed to be most effective amongst the three groups, followed by PC6 electro-acupoint stimulation and PC6 acupressure vs. sham. 

For prevention of vomiting (postoperative 0-24h), PC6 electro-acupoint stimulation vs. sham had the lowest pooled RR, followed by PC6 acupressure vs. sham and PC6 acupuncture vs. no acupuncture. PC6 electro-acupoint stimulation vs. sham seemed to be most effective amongst the 3 groups, followed by PC6 acupressure vs. sham and PC6 acupuncture vs. no acupuncture.

Overall, all modalities seemed to be effective in PONV prevention. Electrical stimulation with ReliefBand or electrodes might be more costly than manual needling, however it is reusable and more effective in some cases. ReliefBand and electrode were less invasive, require minimal training and cost-effective, though local effects such as swelling, erythema[25,26], tenderness or paraesthesia[25] had been reported in few studies with ReliefBand. 

Meta-analysis by Shiao SY, Dibble SL 2006 found that acupressure was more effective in reducing symptoms for adults (pregnant or postoperative) than children, and is as effective and more feasible to use than medications and acupuncture modalities[7]. Study by El-Bandrawy AM et al 2013 showed a significant decrease in nausea and vomiting in patients treated by acupressure in addition to anti-emetic drug; while PC6 TEAS was more effective than acupressure in alleviating PONV after abdominal hysterectomy[46].

#### Acupoint PC-6

For PC6 acupuncture vs. no acupuncture, stimulation of PC6 significantly reduced the number of cases of early vomiting (postoperative 0-6h) and nausea (postoperative 0-24h). However, it seemed not effective for early nausea (postoperative 0-6h) and vomiting (postoperative 0-24h). At postoperative 0-24h, both PC6 acupressure and PC6 electro-acupoint stimulation vs. sham significantly reduced the number of cases of nausea and number of cases of vomiting.

Study by Streitberger K et al 2004[47] on PC6 acupuncture in women undergoing gynaecology and breast surgery showed that differences in incidence of PONV and/or use of anti-emetic rescue were more pronounced in patients having gynaecological surgery (48.9% acupuncture, 67.6% placebo, P=0.07) than breast surgery (38.7% acupuncture, 40.3% placebo, P=0.86). Author concluded acupuncture at PC6 might be effective in patients having gynaecological surgery, but not in patients having breast surgery.

 In a study by Majholm B and Møller AM, 2011[25] using PC6 acupressure vs. sham, no statistical significance was noted for incidence of nausea or vomiting between the treatment and control group in women undergoing breast surgery.

PC6 intervention is simple, inexpensive, and noninvasive with minimal side effects. However, there were limitations with PC6 alone. For example, stimulation of PC6 in eye and breast surgery might not be effective. PC6 combined with other acupoint(s) and use of alternative acupoint(s), such as auricular acupuncture, cupping therapy, catgut embedment, might provide better prospect for prevention and treatment in PONV.

#### P6 combined with other acupoint(s)

Meta-analysis showed that stimulation of PC6 combined with other acupoint(s) significantly reduced the number of cases of PONV compared to control group at postoperative 0-24h.

Stimulation of PC6 combined with other acupoint(s) at postoperative 0-24h had lower pooled RR compared to other acupoint(s) and seemed more effective than the latter. However, the efficacy in prevention of nausea or vomiting alone could not be evaluated due to lack of studies in the former group.

Common acupoints used were ST36 (Zusanli), LI10 (Shousanli) and LI4 (Hegu). ST36 is located along the Stomach Meridian of Foot-Yangming, which function in adjusting qi and blood, food transport and gastrointestinal activity. After surgery it helps to stimulate the relaxation of gastrointestinal contractions, and enhance body resistance. PC6 is located along the Pericardium Meridian of Hand-Jueyin. Stimulation of PC6 help to adjust the endocrine function, release of epinephrine and vasopressin, inhibit gastic acid secretion, regulate gastrointestinal motility, relieve stomach cramps, and has better effect on sympathetic vomiting and anaesthesia-induced nausea and vomiting. Stimulation of PC6 and ST36 produced better and strengthened anti-emesis effect.

Early stimulation of LI10 and ST36 is effective in PONV prevention and treatment in abdominal surgery. Stimulation of ST36 strengthens and helps to regulate the function of spleen and stomach digestion, smooth and clear the function of qi and blood. LI10 is an important acupoint of the Large Intestine Meridian of Hand-Yangming, and directly connected with the large intestine. It is beneficial in the regulation of the flow of qi and blood of the organs and postoperative symptoms of abdominal surgery.

LI4 is located along the Large Intestine Meridian of Hand-Yangming. With combination with ST36, it helps to regulate the stomach to function more smoothly. Stimulation of LI4, PC6 and ST36 effectively inhibit the vagus nerve which helps to stabilise the cardiovascular function, improve anaesthetic effect, enhance analgesia, adjust the autonomic functions of the digestive system, promote gastrointestinal peristalsis and facilitates patients’ recovery.

Study by Yu JM et al 2010[32] on the effect of TEAS on breast radical carcinoma surgery showed that stimulation of LI4 with PC8 and PC6 with TE5 significantly reduced the need of analgesia and number of cases of PONV compared to control (under general analgesia only). It has been demonstrated that acupuncture produces analgesia via the body endorphin system which could be antagonized by naloxone[48]. The analgesic effect of TEAS may be related to its effect in up-regulating plasma beta-endorphin level[32].

#### Other acupoint(s)

Meta-analysis showed that stimulation of other acupoint(s) significantly reduced the number of cases of nausea and/or vomiting in patients at postoperative 0-24h.

Electro-acupuncture at bilateral LI4 with Vit B6 acupoint injection at bilateral ST36[39], bilateral ST36 acupoint injection with Metoclopramide[43], and alternating acupoint massaging were among the effective method used[44].

Lu ZX et al 2009[38] used cupping therapy for PONV prevention among patients undergoing laparoscopy cholecystectomy. Cupping was applied at the patients’ back which consists of Du Mai (GV-, governing vessel) and the Kidney Meridian which helps to regulate the flow of blood and qi to become more smoothly and helps to balance yin and yang.

Yang W et al 2011[40] performed a preoperational catgut implantation at bilateral BL57 on patients undergoing hemorrhoid operation, and found to be significantly more effective than medication in reducing pain, nausea and vomiting. 

Stimulation at acupoints such as large Intestine LI4 (on the hand), Spleen SP6 (on the lower limb), and “back-shu” (paravertebral area) have been shown to have analgesic properties[49].

Auricular acupoint application was found to be effective in reducing pain[42], nausea and vomiting[41,42,45], in adult[42,45] and children[41]. Auricular acupuncture reduced the concentration of 5-HT, which is the main cause of vomiting by acting on the peripheral nerve plexus of the small intestine of the receptor that mediate vomiting[45].

#### Korean Hand acupoint(s)

Boehler M et al 2002[22] found that Korean Hand acupressure on K-K9 (located at middle phalanx of the 4th finger, corresponds to PC6) was effective for reducing PONV in women after minor gynecological laparoscopic surgery.

Other effective Korean hand points (K-K9; K-D2), bladder points (BL10, BL11, BL18-26), spleen points (SP4, SP6), stomach points (ST34, ST36, ST44), and others (GB4, CV12, and others) were found to be as effective as PC6 and sometimes more so[7]. Study by Kim KS et al 2002[50] on capsicum plaster showed the effectiveness of K-D2 in reducing the incidence of PONV after abdominal hysterectomy was comparable to PC6.

### Optimal time and technique of intervention

#### Timing of intervention

Previous meta-analysis indicated that the antiemetic effect of acupuncture require treatment of awake rather than anesthetized patients[51]. Study by White PF et al 2005[52] to deduce the optimal timing of acustimulation for patients undergoing plastic surgery found that perioperative use of ReliefBand (applied for 30min before and 72h after surgery) significantly increased the complete responses (68%) compared to before surgery only (43%) (applied for 30 min before surgery). Median postoperative nausea scores were significantly reduced and patient satisfaction (with quality of recovery and antiemetic management) was significantly higher in the former group. For patients discharged on the day of surgery, time to home readiness was significantly reduced when acustimulation was administered perioperatively (vs. preoperatively). Acustimulation with ReliefBand was most effective in reducing PONV and improving patients' satisfaction with their antiemetic therapy when it was administered after surgery[52].

Systematic review by Holmér Pettersson P and Wengström Y 2012[1] found that acupuncture prior to surgery reduced the incidence of nausea but not vomiting compared to antiemetic prophylaxis alone.

Yentis SM and Vashisht S 1998[53] performed a study on whether antiemetic effect of PC6 acupuncture in preventing PONV is affected by the timing of administration in 50 patients undergoing major gynaecological surgery. Patients were randomly assigned to receive PC6 acupuncture either 5 min before induction of anaesthesia (Group 1), 5 min after induction of anaesthesia (Group 2) or when awake in recovery room post-operatively (Group 3). Results showed no significant differences in the emetic sequelae amongst the three groups, with incidence of vomiting of 29%, 24% and 25% within the first 6h post-operatively. General anaesthesia does not affect the antiemetic action of PC6 acupuncture.

Lee A and Done ML[54] showed that non-pharmacologic techniques (acupuncture, electro-acupuncture, TEAS, acupoint stimulation and acupressure) were more effective than placebo in preventing nausea and vomiting within 6h of surgery in adults, but not in children. Study by El-Bandrawy AM et al 2013 showed that time was an important variable, with significant effects of acupressure in the first 6 h[46]. 

#### Technique of intervention

Rotating, reinforcing-reducing[17,18,37] and rotating[19] were among the common technique used in manual acupuncture. Stimulation was performed for 2min[17,18], 1-2min[37] and 5s[19]. Response of “deqi’ is usually sought to ensure stimulation.

 “Seaband” with pressure stud[21], acupressure seed (2-mm diameter)[22], “Seaband” with beads [23,24,26], “Vital-Band” with stud[25], “ReliefBand”[28] and auricular plaster therapy with Vaccaria seed[41] had been used to exert pressure. In some cases, bead was pressed for 1 min[24] and brief presses of wristband for 30s were performed[25] to achieve stimulation.

It has been suggested that low frequency (2-4Hz) stimulation resulted in the release of endorphin and high frequency (50-200Hz) the release of encephalin[55]. Low frequency stimulation produced analgesia of slower onset but longer duration of time. High frequency stimulation resulted in more rapid onset but shorter duration[55]. Current intensity was usually increased to a degree just less than what caused discomfort or at a degree tolerable to patients.

Tang W et al 2013[56] evaluated the impacts of electro-acupuncture at bilateral PC6 at different frequencies in patients undergoing laparoscopic surgery under general anaesthesia. Patients were randomised into 2Hz electro-acupuncture (group A), 2Hz/100Hz electro acupuncture (group B), 100Hz electro acupuncture (group C) and control (group D). The incidence and severity of PONV in group B was apparently lower than other groups (P<0.01).

Study by Lin JG et al 2002[57] showed that the incidence of nausea during the first 24h after surgery was significantly reduced in low (2Hz) and high (100Hz) electro-acupuncture groups compared to control and sham electro-acupuncture. Both high- and low-frequency electrical stimulation also reduced postoperative analgesic requirement, with best results in high-frequency stimulation. Use of electro-acupuncture also resulted in a decrease in the incidence of opioid-related side effects after lower abdominal surgery.

Acupoint injections combine the effect of both acupoint stimulation and drugs, with Chinese and Western application, and had been proved to be effective in PONV prevention and treatment.

Cupping therapy acts on the meridians and acupoints along the pores and skin, mediate the flow of qi and blood, and balances yin and yang. It is effective in PONV prevention[38].

Catgut embedment involves the theory of acupuncture and needle retention. It forms a complex, soft and durable stimulation, reduces pain and remains longer duration than manual acupuncture. It was found to be significantly more effective than medication in reducing pain, nausea and vomiting[40].

#### Acupoints (unilateral/bilateral)

A trial indicated neither unilateral nor bilateral application of acupressure significantly affected the incidence of nausea and vomiting[58] while another study showed both had mixed effects, although bilateral application seemed to have more consistent complete response (PONV incidence and antiemetic use)[59]. 

#### Needle size and depth of needle insertion

Shorter needles are usually used near the face and eyes, while longer needles are used in more fleshy areas. Thicker needles are often used on more robust patients. 

Needles are usually inserted until “deqi” to achieve stimulation and to a degree which cause least pain and discomfort to patients. 

### Side effects

Overall, acupuncture is safe though there were few reports on local erythema with electro-acupuncture; and redness, erythema, swelling, tenderness and paraesthesia with acupressure bands. The effects were local and no major adverse events followed. 

### Use of rescue anti-emetics

The intervention group seemed to be effective in reducing the use of anti-emetics rescue therapy. 

### Quality evaluation

#### GRADE, CONSORT AND STRICTA FOR TCM

Most of the studies on PC6 combined with other acupoint(s) and other acupoint(s) did not emphasis the details of blinding and allocation concealment. Most of these studies were conducted in mainland China. 

Although high quality evidence doesn’t necessarily imply strong recommendations, and strong recommendations can arise from low quality evidence[13], studies in the future should follow the standard guideline for better quality of evidence.

Future studies should be carried out according to recommendations for better quality of evidence. 

#### Updated from Previous Systematic Reviews[1],[60,61]

1 Efficacy of different type of acupuncture on PC6, PC6 combined with other acupoint(s), and other acupoint(s)) were compared. Studies were further divided according to time of PONV, according to availability of data.
2 Optimal timing, technique of intervention, side effects and use of rescue therapy were considered.
3 Heterogeneity was minimized, with studies varied significantly from others in combination of intervention, study settings or populations were excluded.

#### Other Considerations

For combination of interventions, the order of intervention might need to be considered, as it might affect the efficacy and study outcome. For example, Norheim AJ et al 2010[62] and Liodden I et al 2011[63] performed PC6 acupuncture followed by acupressure in children undergoing tonsillectomy and/or adenoidectomy. Results showed less vomiting in the treatment group compared to control in both studies. On the other hand, Shenkman Z et al 1999[64] performed a study with PC6 acupressure followed by acupuncture, no significant differences in retching and vomiting were demonstrated between the treatment and control group. Hence, type and order of intervention might contribute to the difference in results.

Previous studies on combinations of interventions such as acupuncture with transdermal scopolamine vs. transdermal scopolamine[65], acupoint sticking therapy with massage vs. standard care[66], electro-acupuncture with tropisetron vs. tropisetron[67] at bilateral PC6 and ST36 demonstrated significant better results in intervention compared to control group.

## Limitations

1 There were articles which were not included due to lack of studies to form subgroup under the same type of intervention for meta-analysis. Studies such as laser stimulation[68] and intraoperative stimulation with conventional nerve stimulator[69] also demonstrated the effectiveness of PC6 stimulation on reducing nausea and vomiting compared to control. The use of semi-permanent acupuncture needles at bilateral PC6 was shown to reduce the severity of nausea in the second 24 hours, and have greater effect on patients who had nausea and vomiting after a previous anaesthetic[70].
2 Comparison between PC6 intervention with anti-emetics and efficacy of PC6 intervention at late PONV could not be evaluated due to lack of studies.
3 Studies in the PC6 combined with other acupoint(s) and other acupoint(s) could not be further subgrouped according to type of acupuncture and time of PONV due to lack of studies.

## Conclusion

Acupuncture for prevention and treatment of PONV is worth popularising for its efficacy, safe, cost effectiveness and benefits. It also has analgesic effects and could serve as pain relief.

Besides PC6, PC6 combined with other acupoint(s) and other alternative acupoint(s) might be beneficial in prevention and treatment of PONV, the evidence justifies future high-quality studies. 

## Supporting Information

Checklist S1 **PRISMA Checklist.**
(DOCX)

Table S1 **Data summary and GRADE of the 16 studies included in meta-analysis for PC6 (**A**).** Data summary and GRADE of the 6 studies included in meta-analysis for PC6 combined with other acupoint(s) (B). Data summary and GRADE of the 8 studies included in meta-analysis for other acupoint(s) (C).
(DOCX)
