# Supplementary material for: The Effectiveness of Acupuncture in Prevention and Treatment of Postoperative Nausea and Vomiting - A Systematic Review and Meta-Analysis
Source: PLoS One. 2013 Dec 13;8(12):e82474. doi: 10.1371/journal.pone.0082474 (PMC3862842; doi:10.1371/journal.pone.0082474)
Supplement: Table S1 — Data summary and GRADE of the 16 studies included in meta-analysis for PC6 (A). Data summary and GRADE of the 6 studies included in meta-analysis for PC6 combined with other acupoint(s) (B). Data summary and GRADE of the 8 studies included in meta-analysis for other acupoint(s) (C). (DOCX) [file pone.0082474.s002.docx]

Table S1A. Data summary and GRADE of the 16 studies included in meta-analysis for PC6

| **Author,**  **Year**  **(Country)** | **Study Population** | **Type and duration**  **of surgery** | **Type and duration of anaesthesia** | **Experimental**  **events** | **Control**  **events** | **Rescue anti-**  **emetics** | **Side**  **effects** | **GRADE** |
| --- | --- | --- | --- | --- | --- | --- | --- | --- |
| Dundee JW,  1986  (Ireland)[16] | Fit women  16-60 yo,  40-80 kg | Minor  gynaecological  surgery  (7-12)min | Methohexitone-  nitrous-oxide-  oxygen with no volatile supplements | Acupuncture  (n=25) | No acupuncture  (n=25) | N/A | Nil | ⊕⊕ΟΟ  LOW |
| Lv H,  2012  (China)[17] | Adult  I: M/F 12/13  65-81 (75.8)yo  C:M/F 11/14  66-79 (76.4)yo | Hemi-  arthroplasty | General  anaesthesia | Acupuncture  (n=25)  bilateral | No acupuncture (n=25) | Metoclopramide 20mg i/m | N/A | ⊕⊕ΟΟ  LOW |
| Ouyang MW,  2009  (China)[18] | Adult  I: M/F 37/13  28-77 yo,  (55.4±22.5) yo  (62.5±18.5) kg  C: M/F 31/19  45-79 yo,  (56.7±26.4) yo  (64.7 ±34.1) kg | Laparoscopic  gastrointestinal  surgery  I: (124.6 ±21.0)  min  C:(139.7 ±31.6)  min | Combined infusion and inhalation anesthesia (PCA)  I:(159.3±23.1)  min  C:(164.5±37.5)  min | Acupuncture  (n=50)  bilateral | No acupuncture (n=50) | Metoclopramide 20mg i/v | Nil | ⊕⊕⊕Ο  MODERATE |
| Al-Sadi M,  1997  (UK)[19] | Women  I:(34.2±6.1) yo,  (61.7±9.7) kg  C:(35.8±8.0 )yo,  (64.0 ±9.6) kg | Day case gynaecology  laparoscopic | General  anaesthesia | Acupuncture  (n=40)  bilateral | No acupuncture (n=41) | Ondansetron i/v  I: 10 doses  C: 13 doses | Nil | ⊕⊕⊕⊕  HIGH |
| Yentis SM,  1991  (Canada)[20] | Children  I:2-11 (6.7) yo  C: 2-10 (5.7) yo | Elective  tonsillectomy | General  anaesthesia  I: (24±7) min  C:(26±7) min | Acupuncture  (n=23)  left unilateral | No acupuncture  (n=22) | Dimenhydrinate 1mg/kg i/m | N/A | ⊕⊕⊕Ο  MODERATE |
| Allen DL,  1994  (England)[21] | Women  I:(41.6±7.4) yo, (70.7±16.4) kg  C:(44.2±11.2)yo, (73.7±17.1) kg | Laparotomy for  major gynaecological surgery | Combined infusion and inhalation anesthesia (PCA) | Acupressure  (n=23)  “Seaband” with pressure stud on the dominant arm | Sham acupressure  (n=23)  dorsum of wrist of the dominant arm | Prochlorpera-zine  1.25mg i/m qid prn  I: 22 doses  C: 33 doses | N/A | ⊕⊕ΟΟ  LOW |
| *Boehler M,  2002  (Austria)[22] | Women  I:(35.4±9.6)yo,  (60.9±10.1) kg  BMI:(22.3±2.9)  C:(38.0±10.9)yo  (63.9±10.3) kg  BMI:(23.1±3.1) | Minor gynecological  laparoscopic surgery  I:(47.1±13.5)min  C:(51.1±16.7)min | General  anaesthesia | Acupressure  (n=40)  with acupressure seed,  bilateral  middle phalanx of fourth finger | Sham acupressure  (n=40)  bilateral  ulnar sides  of 5^th^ finger | Tropisetron 2-5 mg i/v | Nil | ⊕⊕⊕Ο  MODERATE |
| Harmon D,  2000  (Ireland)[23] | Women  I:18-38 (30) yo,  (78±12) kg  (163±6) cm  C:19-40 (32) yo,  (77±11) kg  (160±7) cm | Elective caesarean  section | Spinal anaesthesia | Acupressure  (n=47)  using  acupressure bands with spherical beads,  right unilateral | Sham acupressure (n=47)  dorsal right forearm | Ondansetron 4mg i/v during surgery or cyclizine 50mg i/m tds after surgery)  Ondansetron:  I: 0-4 (0.08) mg  C:0-4 (0.5) mg  P=0.002  Cyclizine:  I: 0-50 (13.0)mg  C:0-100 (25.0) mg  P=0.002 | No major side effect | ⊕⊕⊕⊕  HIGH |
| Klein AA,  2004  (Canada)[24] | Adult  I:M/F 68/6  (62±10) yo,  (79±12) kg  (170±8) cm  C:M/F 60/17  (63±8) yo,  (79±12) kg  (169±9) cm | Cardiac surgery  I: (3.5±0.6) h  C: (3.3±0.7) h | General  anaesthesia  I:(4.3±0.7)h  C:(4.1±0.7)h | Acupressure (n=75) “SeaBand” with spherical beads,  bilateral | Sham  Acupressure  (n=77)  without bead,  bilateral | Dimenhydrinate  50mg i/v  I: 22(29%)  C:27(35%)  no of patients (%)  P=0.45 | N/A | ⊕⊕⊕Ο  MODERATE |
| Majholm B,  2011  (Denmark)[25] | Women  I:49-68 (62)  yo  C:55-69 (63)  yo | Breast surgery  I: 25-350 (107.5) min  C:12-295 (105.0) min | Total i/v  anaesthesia | Acupressure  (n=67) “Vital-Band” with stud,  ipsilateral to the site of anaesthesia | Sham acupressure  (n=67)  dorsum of  forearm,  ipsilateral to the site of anaesthesia | According to consensus guideline Gan TJ et al, Anesth Analg 2003; 97:62–71.  I:16, C:16  P=0.834 | 1/3 reported redness, swelling, tender-ness | ⊕⊕⊕Ο  MODERATE |
| Turgut S,  2004  (Turkey)[26] | Women  I:(45±7) yo  (65±11) kg  (159±7) cm  C:(47±7) yo  (68±11) kg  (161±6) cm | Elective gynaecological  surgery (TAHBSO)  I:(81±30)min  C:(81±25)min | Combined infusion and inhalation anesthesia (PCA) | Acupressure  (n=51)  “Seaband” with plastic bead,  bilateral | Sham acupressure  (n=51)  dorsal surface of forearm | Metoclopramide  10mg i/v  I: 13/19*  C:33/48  (patients/doses)  *P=0.001 | 1 patient had swelling and erythema | ⊕⊕⊕Ο  MODERATE |
| Amir SH,  2007  (India)[27] | Adult  I:M/F 12/8 (17.95±8.25)yo, (37.8±14.71) kg  C:M/F 8/12 (21.1±7.48)yo, (40.4±9.45) kg | Middle ear surgery | General  anaesthesia  I:(113.25±  30.27)min C:(104.5±  35.68)min | Electro-  acupuncture  (n=20)  4Hz | Sham  (n=20) | Ondansetron 4mg i/v  I: 6/11*  C:16/34  (patients/doses)  *P<0.01 | Local erythema in 15% of cases | ⊕⊕⊕Ο  MODERATE |
| Habib AS,  2006  (USA)[28] | Women  I:(30±6) yo,  (90±19) kg  (165±8) cm  C:(30±6) yo,  (90±20) kg  (164±6)cm | Caesarean  delivery  I:(65±28)min  C:(68±24)min | Spinal anaesthesia | Trans-cutaneous  electrical  acupoint  stimulation  (n=47)  with active ReliefBand at the dominant hand | Sham  (n=44)  with active ReliefBand at dorsum of the wrist of the dominant hand | Ondansetron  4mg i/v  Intraoperative  I: 11(23%)  C:8(18%)  P=0.54  Postoperative  I:16(34%)  C:17(39%)  P=0.65 | N/A | ⊕⊕⊕⊕  HIGH |
| Liu YY,  2008  (China)[29] | Adult  I: M/F 26/22  (42±18) yo,  (61±12) kg  C: M/F  25/23  (40±19) yo,  (58±13) kg | Laparoscopic  cholecystectomy  I:(108±38)min  C:(115±36)min | General  anaesthesia  I: (136±37)  min  C: (142±34)  min | Active electrical  stimulation  (n=48)  left unilateral,  with  conventional  peripheral nerve stimulator (PNS) train-of-four (TOF) mode  2-100Hz, 50ms, 0.5-4mA | Sham  (n=48)  inactive device at  left PC6 | Ondansetron  4mg i/v  I:9(18.7)*  C:18(37.5)  case(%)  *P<0.05 | Nil | ⊕⊕⊕Ο  MODERATE |
| Wang XQ,  2010  (China)[30] | Adult  I:(43±11) yo,  (59±12) kg  (163±6) cm  C:(41±9) yo,  (60±9) kg  (161±5) cm | Supratentorial  craniotomy  I:(213±45)min  C:(222±52)min | General  anaesthesia | Trans-  cutaneous  electrical acupoint  stimulation  (n=40)  alternating  2Hz to 100Hz,  right unilateral | Sham  (n=40)  at a non acupoint site,  dorsal surface of the forearm | Metoclopramide  10mg i/v  I:6/11  C:11/8  (patients/doses)  No significant differences between groups | N/A | ⊕⊕⊕Ο  MODERATE |
| Xu M,  2012  (China)[31] | Adult  I: M/F 23/37  (46±13) yo,  (62±10) kg  (165±8) cm  C:M/F 24/36  (47±11) yo,  (61±11) kg  (166±8) cm | Elective  infratentorial  craniotomy | General  anaesthesia  I:(6.5±1.8) h  C:(6.6±1.7) h | Trans-  cutaneous  electrical acupoint  stimulation  (n=60)  alternating 2Hz to 100 Hz at 2mA  at PC6 of the dominant side | Sham  (n=59)  at PC6 of the dominant side | Metoclopramide  10mg i/m  I:11.14  C:18/22  (patients/doses)  Overall requirements were similar between groups | Nil | ⊕⊕⊕⊕  HIGH |

Abbreviations: I – Intervention

C – Control

* Korean hand acupuncture point K-K9 which is corresponds to the Chinese acupoints PC6

Table S1B. Data summary and GRADE of the 6 studies included in meta-analysis for PC6 combined with other acupoint(s)

| **Author, Year**  **(Country)** | **Study Population** | **Type and duration**  **of surgery** | **Type and duration of anaesthesia** | **Experimental**  **events** | **Control**  **events** | **Rescue anti-**  **emetics** | **Side**  **effects** | **GRADE** |
| --- | --- | --- | --- | --- | --- | --- | --- | --- |
| Yu JM,  2010  (China)[32] | Women  20-70 yo,  45-70 kg | Radical surgery for breast cancer | General anaesthesia | Trans-  cutaneous electrical acupoint stimulation(TEAS)  (n=30)  2Hz/100Hz  5-10mA  LI4 with PC8,  PC6 with TE5 | No acupuncture  (n=30) | N/A | N/A | ⊕⊕ΟΟ  LOW |
| Liu SF,  2010  (China)[33] | Adult  I: M/F 25/25  27-77 (50.56  ± 11.76)yo  C:M/F 23/27  24-78 (50.48  ±14.47)yo | Endoscopic surgery | Not mentioned | Trans-  cutaneous electrical acupoint stimulation(TEAS  (n=50)  at bilateral LI4, PC6 +  relaxation therapy | Psychological counseling  (n=50) | N/A | N/A | ⊕⊕ΟΟ  LOW |
| Li J,  2011  China[34] | Adult  M/F 56/21  30-68 (49) yo | Femoral artery chemo  embolization  (TACE) | Not mentioned | Acupoint injection with Metoclopramide  hydrochloride 20mg dexa-methasone 5mg  (n=38)  bilateral PC6 (0.5ml), ST36 (1ml) | Azasetron  hydrochloride  injection  (n=39) | N/A | Nil | ⊕⊕ΟΟ  LOW |
| Wang H,  2004  (China)[35] | Women  I: (57.50±6.29)yo  (57.00±8.80)kg  (155.75±6.81)cm  C:(56.68±6.80)yo  (53.86±7.49)kg  (153.78±5.87)cm | Hysterectomy and bilateral oophorectomy | Continuous epidural block via L1-L2 | Electro-  acupuncture  (n=30)  50-100Hz  bilateral PC6, LI4, ST36 | No  acupuncture  (n=30) | N/A | N/A | ⊕⊕ΟΟ  LOW |
| Yuan J,  2000  (China)[36] | Women  31-48 yo | Abdominal total hysterectomy | Epidural anaesthesia  via T12-L1 and L3-L4 | Electro-acupuncture  (n=25)  wave density (D-D), 16- 50Hz, 10-15mA  bilateral PC6, LI4, ST36 | No  acupuncture  (n=25) | N/A | Nil | ⊕⊕ΟΟ  LOW |
| Pei Y,  2010  (China)[37] | Adult  I: M/F 60/30  (47.8) yo,  (67.4) kg  C:M/F 59/31  (46.9) yo,  (70.5) kg | Abdominal and pelvic surgery  I:(126) min  C:(134) min | General anaesthesia | Acupuncture  (n=90)  main acupoint: bilateral PC6, LI4, BL10, GB34, ST36, SP4, CV12,  supplementary  acupoint: LV3, SP6, SP9, ST40 | No intervention  (n=90) | N/A | No major side effects | ⊕⊕ΟΟ  LOW |

Abbreviations: I – Intervention

C – Control

Table S1C. Data summary and GRADE of the 8 studies included in meta-analysis for other acupoint(s)

| **Author, Year**  **(Country)** | **Study Population** | **Type and duration**  **of surgery** | **Type and duration of anaesthesia** | **Experimental**  **events** | **Control**  **events** | **Rescue anti-**  **emetics** | **Side**  **effects** | **GRADE** |
| --- | --- | --- | --- | --- | --- | --- | --- | --- |
| Lu ZX,  2009  (China) [38] | Adult  M/F 35/25  25-68 (46.4) yo | Laparoscopic  cholecystec-  tomy | General anaesthesia | Flash fire cupping  (n=30)  BL11-BL23, BL41-BL52  (at 6h post-operative)  GV14-GV4  (at 24h post-operative) | Standard care  (n=30) | N/A | N/A | ⊕⊕ΟΟ  LOW |
| Yao AJ,  2012  (China)[39] | Women  I:(29.80±5.45)yo,  (56.87±8.77) kg  (149.03±5.64)cm  C:(28.87±6.29)yo,  (53.37±8.09) kg  (151.57±5.43)cm | Cesarean  section | Epidural anaesthesia L3-L5 | Electro-acupuncture  10-50Hz, 1-2mA at  bilateral LI4 +  acupoint injection with 50mg Vit B6 at bilateral ST36  (n=30) | No  intervention  (n=30) | N/A | N/A | ⊕⊕ΟΟ  LOW |
| Yang W,  2011  (China)[40] | Adult  I: M/F 34/26  (42.1±11.5) yo  C:M/F 33/27  (43.6±12.1) yo | Hemorrhoid  surgery | Local nerve  block anaesthesia | Catgut embedment  (n=60)  bilateral BL57 | 2 tablets Tramadol  hydrochloride  50mg each  (n=60) | N/A | N/A | ⊕⊕ΟΟ  LOW |
| Zeng QL, 1999  (China)[41] | Children  1-11 (6.57) yo | Paediatric surgery  10-250  (105) min | General anaesthesia | Auricular plaster therapy with Vaccaria seed  (n=56)  bilateral CO13, C04, AT(brain), TF4 | No intervention  (n=47) | N/A | Nil | ⊕⊕ΟΟ  LOW |
| Wetzel B,  2011  (German)[42] | Adult  I:M/F 26/34  (66±10) yo,  (84±14) kg  BMI (30±4)  C:M/F 24/36  (67±8) yo,  (82±15) kg  BMI (29±5) | Elective unilateral THA for degenerative osteoarthritis  I:(80±28)min  C:(82±28)min | General anaesthesia  I:(119±29)min  C:(120±30)min | Auricular acupuncture  (n=57)  ipsilateral to the surgery site at MA-AH4 (AH5) (Hip), MA-TF1(TF4)  (Shenmen), MA-IC1 (CO14) (Lung) | Sham acupuncture  (n=59)  ipsilateral to the surgery site at 3 non acupoints of the helix | N/A | Nil | ⊕⊕⊕Ο  MODERATE |
| Zeng ZD,  2011  (China)[43] | Adult | Endoscopic surgery | Local anaesthesia with oral Dyclonine mucilage | Acupoint injection with Metoclopra-mide 10mg  (n=30)  bilateral ST36 | Standard care  (n=30) | N/A | N/A | ⊕⊕ΟΟ  LOW |
| Li GP,  2013  (China)[44] | Adult | Laparoscopic  cholecystec-tomy | General anaesthesia | Acupoint massage  (n=65) alternating acupoint massaging  at bilateral  ST36 | Standard care  (n=65) | N/A | N/A | ⊕⊕ΟΟ  LOW |
| Wang ZY,  2009  China[45] | Women | Cesarean section | Epidural anaesthesia  Via L2-L3 | Auricular acupuncture  (n=15)  main acupoint at right TF4, AT(brain), CO18, with supplementary acupoint at  TF5, TF(Uterus) | No intervention (n=15) | N/A | N/A | ⊕⊕ΟΟ  LOW |

Abbreviations: I – Intervention

C – Control
